# Evaluation of serine protein kinase HPrK as an antibacterial target in gram-positive bacteria and mycoplasmas

**DOI:** 10.1128/spectrum.03977-25

**Published:** 2026-03-17

**Authors:** Hengfei Yan, Jiajia Xu, Tingting Jiang, Siyang Lu, Yuling Liu, Ying Sun, Hongyu Wu, Qiao Hu, Lu Li, Qi Huang, Tengfei Zhang, Rui Zhou

**Affiliations:** 1 State Key Laboratory of Agricultural Microbiology, Hubei Hongshan Laboratory, College of Veterinary Medicine, Huazhong Agricultural University 47895, Wuhan, China; 2 Key Laboratory of Prevention and Control Agents for Animal Bacteriosis (Ministry of Agriculture and Rural Affairs of China), Hubei Provincial Key Laboratory of Animal Pathogenic Microbiology, Institute of Animal Husbandry and Veterinary, Hubei Academy of Agricultural Sciences 117996, Wuhan, China; 3 International Research Center for Animal Disease (Ministry of Science & Technology of China), Wuhan, China; 4 The Cooperative Innovation Center of Sustainable Pig Production, Wuhan, China; Universita degli Studi di Bari Aldo Moro, Bari, Italy

**Keywords:** *Streptococcus suis*, HPrK, essential protein kinase, antimicrobial target, inhibitor screening

## Abstract

Antimicrobial resistance (AMR) poses a serious threat to global public health. To tackle this challenge, identifying novel effective antibacterial targets and antibiotics is crucial. The serine kinase HPrK, widely present in gram-positive bacteria and mycoplasmas, primarily regulates carbon metabolism and virulence factor expression. In a previous study, we failed to knockout the *hprK* gene using the conventional homologous recombination method in the zoonotic *Streptococcus suis*. Here, we employed an anhydrotetracycline (ATc)-inducible promoter (AiP) and *hprK* fusion to replace its original expression cassette in the *S. suis* genome. However, in the absence of ATc, mutations in the AiP elements resulted in ATc-independent expression of HPrK, suggesting that HPrK is essential for *S. suis* and a potential antimicrobial target. Accordingly, a high-throughput inhibitor screening assay based on the kinase activity of HPrK was designed, and an inhibitor compound CDK9-IN-2 was identified. Molecular docking and bio-layer interferometry revealed that CDK9-IN-2 targets highly conserved residues D242, D249, and N281 on HPrK protein in several species of gram-positive bacteria. CDK9-IN-2 exhibited significant antibacterial effects against multidrug-resistant *S. suis* clinical isolates and various species of gram-positives and mycoplasmas with a minimum inhibitory concentration (MIC) of 16 to 32 μg/mL. Its therapeutic effect on *S. suis* infection was further proved in a *Galleria mellonella* larval infection model. Collectively, HPrK is a conserved kinase in gram-positives and mycoplasmas, and its essentiality is confirmed in *S. suis*. Based on this conserved target HPrK, an inhibitor with a broad antibacterial activity has been identified, suggesting HPrK is a promising antimicrobial target for antibacterial drug innovation.

**IMPORTANCE:**

Antimicrobial resistance (AMR) is a critical global health challenge, highlighting the need for new antibiotic targets. HPrK is a serine kinase that is important in regulation of carbon metabolism and virulence in gram-positive bacteria and mycoplasmas. In this study, we validated that HPrK was an essential gene in *S. suis* and could be used as a promising target for antibacterial drug development. Based on HPrK, we identified the kinase inhibitor CDK9-IN-2, which exhibits significant antibacterial activity against multidrug-resistant *S. suis* isolates and various gram-positive species and mycoplasmas. These findings indicated HPrK as a viable target for novel antimicrobial agents, advancing strategies to combat antimicrobial resistance.

## INTRODUCTION

Antimicrobial resistance (AMR) has emerged as a global challenge for public health, presenting severe threats to both human and animal health (1, 2). In the last few decades, few novel antibiotics have been discovered and applied, further exacerbating the AMR challenges (3). Therefore, it is urgent to identify innovative antimicrobial targets for the discovery of novel antibiotics (4). Traditional antimicrobial agents primarily target the key enzymes or structural components in the basic physiological processes directly, such as the synthesis of cell wall (5), protein (6) and nucleic acid (7), and membrane function (8). However, high selective pressure also accelerates the generation of resistant bacteria. Recently, with further study on the pathogenicity and adaptation mechanism of pathogens, significant advancements have been made in discovering novel targets, such as virulence factors (9), biofilm formation (10, 11), metabolic pathways (12), and signal transduction and regulation systems (13).

Carbon metabolism is the central energy-generating pathway in bacteria, involving the utilization of diverse carbohydrates, including monosaccharides (e.g., glucose, fructose, and mannose), disaccharides (e.g., sucrose and lactose), and organic acids (e.g., acetate and lactate) (14). Carbon catabolite repression (CCR) is the key regulatory mechanism for carbon utilization and controls the expression of 5%–10% of genes in many bacteria (14, 15). In gram-positive bacteria, CCR is primarily mediated by the CcpA regulator (catabolite control protein A), which is in complex with phosphorylated HPr (HPr-Ser-P) to repress gene expression (16).

HPrK (HPr kinase/phosphatase) is a dual-functional kinase and phosphatase, which controls the phosphorylation and dephosphorylation of the HPr protein. In the presence of a preferred carbon source (e.g., glucose), HPrK phosphorylates HPr at Ser46, generating HPr-Ser-P. The HPr-Ser-P and CcpA complex binds to the catabolite responsive element (*cre*) sites to repress the expression of secondary/alternative carbon source metabolism-related genes, such as *galKT*, *malQ1*/*malQ2*, and *dhaKL*s. Conversely, upon depletion of the preferred carbon source, HPrK dephosphorylates HPr-Ser-P, resulting in de-repression and facilitating the utilization of other alternative carbon sources (17, 18). Beyond its role in carbon metabolism, HPrK also participates in the pathogenicity of bacteria. In *Listeria monocytogenes*, HPrK indirectly influences PrfA (a transcriptional activator) activity by modulating HPr phosphorylation (19). Furthermore, in *Clostridium difficile*, HPrK directly controls the expression of toxin genes *tcdA* and *tcdB* (20). Therefore, HPrK not only plays important roles in carbon metabolism but also in virulence.

*Streptococcus suis* is a globally distributed gram-positive facultative anaerobic coccus, which poses significant threats to both swine production and public health (21). Previous studies found that CCR played an important role in the regulation of carbon metabolism genes during glucose starvation, and lots of virulence factors were controlled by CcpA in *S. suis* (17, 22). To further understand the function of CCR in *S. suis*, we have tried to knock out the *hprK* gene by traditional homologous recombination, but it failed. In *Staphylococcus xylosus* (23) and *Bacillus subtilis* (24), *hprK* deletion caused severe growth defects, while in *Streptococcus pneumoniae*, *hprK* seemed unable to be deleted (25). Therefore, we supposed that HPrK may be essential for the survival and/or proliferation of *S. suis* and could be a potential antimicrobial target.

In this study, an anhydrotetracycline (ATc)-inducible expression system (26) was employed to rigorously validate the essentiality of the serine kinase HPrK in *S. suis*, and an HPrK-specific inhibitor compound was screened out with significant antibacterial effects against multidrug-resistant *S. suis* clinical isolates and various species of gram-positives and mycoplasmas. The conserved binding sites of the inhibitor on the HPrK enzyme could explain its broad antimicrobial activity. Therapeutic potential on *S. suis* was confirmed in a *Galleria mellonella* larval infection model.

## MATERIALS AND METHODS

### Bacterial strains and growth conditions

The bacterial strains used in this study are listed in Table S3. *S. suis* SC19 is a highly virulent strain isolated from a diseased pig in Sichuan, China, in 2005 (27). *S. suis* and its derivatives were cultured at 37°C on Tryptic Soy Agar (TSA) or in Tryptic Soy Broth (TSB; BD Biosciences, USA) supplemented with 5% inactivated newborn bovine serum (Sijiqing Biotech, Hangzhou, China). The *Escherichia coli* strains DH5α and BL21 (DE3) were grown in Luria-Bertani (LB) broth or on LB agar plates (OXOID, UK) at 37°C. *Mycoplasma* strains were cultured in commercial *Mycoplasma* culture media (Haibo Biotech, Qingdao, China) supplemented with 10% inactivated pig serum (Pingrui Biotech, Zhengzhou, China) at 37°C in a 5% CO₂ environment. All other strains used in the antibacterial activity experiments were cultured at 37°C in Cation-Adjusted Mueller-Hinton Broth (CAMHB; Haibo Biotech, Qingdao, China) or on Mueller-Hinton Agar medium (MHA; OXOID). *S. pneumoniae* was cultured at 37°C in a 5% CO_2_ environment on MHA enriched with 5% sheep blood (Baibo Biotech, Jinan, China) or in CAMHB supplemented with 5% lysed horse blood (Baibo Biotech, Jinan, China). The isolation locations and antibiotic profiles of the *S. suis* clinical strains are given in Table S3.

### Construction of inducible deletion and fluorescent strains

To insert an anhydrotetracycline (ATc)-inducible *hprK*, the ORF of *hprK*, an ATc-inducible promoter (AiP) sequence (26), and the up- and down-stream homologous regions of the ectopic insertion site were amplified and cloned into pSET4s to generate pSET4s-*Pe-tetO-hprK*. This plasmid was electroporated (2.5 kV, 25 mF, and 200 Ω) into *S. suis* SC19 to create the ectopic inducible *hprK* strain (eiHPrK). The native *hprK* gene in the eiHPrK strain was deleted by the traditional homologous recombination, as previously reported (28), and defined as idHPrK. To induce gene silencing, the strains cultured in TSB supplemented with 5% fetal bovine serum (FBS) and 40 ng/mL ATc were washed 5 times with ATc-free medium and resuspended in fresh TSB supplemented with 5% FBS. To evaluate the activities of mutated promoters, different mutated AiPs were fused to the *gfp* gene in pSET7 (Fig. S2), and the resulting plasmids were electroporated into *S. suis* SC19. The AiP sequence is available in GenBank under accession no. ON391044. Throughout the mutant construction, 40 ng/mL ATc was maintained to ensure *hprK* expression. Primers are listed in Table S1.

### Growth curves

The idHPrK was cultured in TSB with 5% FBS and 40 ng/mL ATc to OD_600_ = 0.4 and then washed 5 times with ATc-free or ATc-containing fresh media and diluted to the same OD_600_ (OD_600_ = 0.01). As a control, *S. suis* SC19 was cultured in TSB with 5% FBS to OD_600_ = 0.4 and then washed 5 times with fresh medium and diluted to OD_600_ = 0.01. OD_600_ was measured every 30 min using the Biophotometer (Eppendorf). The experiment was repeated 3 times. Statistical data were modeled using a Gompertz nonlinear regression model, and the estimated parameters were compared across groups using one-way ANOVA, followed by Dunnett’s multiple-comparison test.

### RNA extraction and quantitative real-time reverse transcription PCR

RNA was extracted after culturing the bacterial cells for 3 h (OD_600_ = 0.4–0.5) following the washing. RNA extraction and quantitative real-time reverse-transcription PCR (qRT-PCR) were performed as previously described (29). The bacteria were incubated in 100 µL lysozyme (50 mg/mL) at 37°C for 5 min, and then 800 μL TRIzol (RNAiso Plus, Takara) was added and incubated for 5 min. Subsequently, 200 µL of chloroform was added per mL of TRIzol, and samples were vortexed vigorously. The supernatant was added to an equal volume of isopropanol. The RNA precipitation was then washed with an equal volume of absolute ethanol. The purity and integrity of RNA were verified by 5% agarose gel electrophoresis and Experion Automated Electrophoresis System (Bio-Rad Laboratories).

RNA from each sample was converted to cDNA using the HiScript III RT SuperMix for qPCR (Vazyme). cDNA was diluted 100-fold and subjected to real-time PCR amplification using AceQ qPCR SYBR Green Master Mix (Vazyme) with specific primers (Table S1). Each group comprises four technical replicates. Data were normalized to a reference housekeeping gene (16S rRNA).

### Protein expression and purification

The ORFs of *hprK* and *hpr* genes were cloned into pET28a to generate pET28a-His-HPrK and pET28a-His-HPr, respectively. *E. coli* BL21(DE3) harboring pET28a-His-HPr or pET28a-His-HPrK were grown to the mid-log phase at 37°C and induced with 1 mM isopropyl β-D-1-thiogalactopyranoside (IPTG) for 13 h at 18°C. His-tagged HPr and HPrK were purified from the supernatant using Ni-NTA affinity chromatography (GE Healthcare, cat. no. 10271899), concentrated by ultrafiltration, and stored at −80°C.

### Fluorescence microscopy observation

*S. suis* cultures (5 mL TSB) were grown to OD_600_ = 0.5 at 37°C and then 1:10 diluted in fresh TSB and incubated for 30 min. After adding 40 ng/mL ATc, cultures were incubated for an additional 15 min at 37°C. Cells were harvested and washed twice with phosphate-buffered saline (PBS) and then resuspended in 75 µL PBS. A 20 µL aliquot of each cell was mounted on a coverslip using 1% agarose and imaged using an Olympus BX53 fluorescence microscope. Images were captured at a magnification of 100×, with bright-field exposure set at 625 μs and GFP (excitation/emission: 488/540 nm) exposure at 260 ms.

### Peptidoglycan staining and membrane staining

Peptidoglycan was stained with 200 µM HCC-amino-D-alanine (HADA; WuXi AppTec), and membranes were stained with 20 µg/mL AF647 (Thermo Fisher Scientific, Cat. No. A20006). *S. suis* cells were incubated with HADA at 37°C for 20 min and resuspended in PBS. Then, AF647 was added and incubated at 37°C for 30 min, followed by PBS washes twice. The stained cells were mounted on slides with 1% agarose and imaged using a Nikon NIS-Elements microscope. AF647 fluorescence was detected (excitation: 647 nm; emission: 663–738 nm; 50 ms exposure), and HADA fluorescence was visualized in the 405 nm channel (40 ms exposure).

### High-throughput screening (HTS) of HPrK inhibitors

A kinase inhibitor library containing 1,133 compounds (HY-LD-000001801; MedChemExpress) was used for screening the HPrK inhibitors. Each reaction was carried out in a 10 μL mixture at 37°C. The mixture contained 150 mM NaCl, 10 mM MgCl₂, 50 mM Tris-HCl (pH 8.0), 100 µM HPr, 2 µM HPrK, 80 µM ATP, and 0.5 mM tested compound, and the reaction was performed in 384-well plates. Each reaction was performed in triplicate. The positive control contained all the components except the tested inhibitors, and the negative control contained all the components except HPrK and the tested inhibitors. After 1 h incubation, 10 µL of Kinase-Glo reagent was added and kept for 10 min. Subsequently, the chemiluminescence value was measured in Relative Luminescence Units (RLU), which represents the photon signal converted to a relative luminescence value, as detected by the photomultiplier tube of the BMG FLUOstar Omega fully automated multifunctional plate reader (Germany). The percentage of inhibition was calculated as [(RLU_X_ − RLU_P_) / (RLU_N_ – RLU_P_)] × 100%. The RLU_X_ was the RLU value after treatment with the tested inhibitors, the RLU_P_ was the RLU value of the positive control, and RLU_N_ was the RLU value of the negative control (30, 31).

### *In silico* docking

The structures of wild-type and mutant HPrK proteins were predicted using the Chai-1 AI model (https://www.chaidiscovery.com/blog/introducing-chai-1). Molecular docking simulations were performed with HPrK, CDK9-IN-2, and ATP. Optimal docking poses were visualized as three-dimensional protein-ligand complexes using PyMOL (https://www.schrodinger.com/). Amino acid sequence alignments were generated with MEGA X (https://www.megasoftware.net/) and visualized using ESPript 3.0 (http://espript.ibcp.fr/ESPript/ESPript/) (32). The SMILES formats of CDK9-IN-2 and ATP were ClC1=CN=C(N[C@@H]2CCC@@HCC2)C=C1C3=NC(NCC4=CC(F)=CC=C4)=CC=C3 and C1=NC(=C2C(=N1)N(C=N2)[C@H]3C@@HO)N, respectively.

### Bio-layer interferometry (BLI) binding kinetics assays

BLI measurements were performed on the Octet RED 96 system (ForteBio, Shanghai, China). Super-streptavidin (SSA) biosensors were equilibrated in PBS for 60 s and then loaded with 200 µL of biotinylated HPrK protein solution for 300 s. The protein-coupled biosensors were immersed in small-molecule solutions for 120 s, and the association kinetics were recorded. Dissociation was monitored for 90 s in PBS buffer. Data were analyzed using ForteBio Data Analysis Software (v11.0), and binding kinetics were fitted using a 1:1 binding model to calculate K_D_ and R² values.

### Antibacterial activity analysis

MIC and minimum bactericidal concentration (MBC) were determined following the guidelines outlined by the Clinical and Laboratory Standards Institute (33, 33). For the tested bacteria, 1 × 10⁶ CFU/mL of bacterial suspensions were used. For *mycoplasma* species, 1 × 10⁴ CCU/mL of suspensions were used. The MIC was determined by using 2-fold serial dilutions of the tested compound (0.0625–128 μg/mL). The MBC was determined by sub-culturing the test dilutions on the plates. The compound was dissolved in dimethyl sulfoxide (DMSO) and then sterilized with a 0.22-mm syringe filter (Millipore, USA). *S. suis* clinical isolates (Table S3), purified by TSA streaking and identified by PCR, were also used for the MIC and MBC testing. All the above experiments were performed in triplicate.

### *Galleria mellonella* larval infection model

The efficacy of CDK9-IN-2 against *S. suis* infection was assessed using the *G. mellonella* larval model (34). A total of 40 larvae were divided into 4 groups. Among them, 3 groups were injected with 1 × 10⁶ CFU of *S. suis* (left proleg). At 1 h post-infection, they were treated with 0, 8, or 16 mg/kg of CDK9-IN-2 (right proleg). Another control group received physiological saline. The survival of each group was monitored every 6 h for 72 h.

## RESULTS

### Construction and characterization of an ATc-inducible *hprK*-deletion strain of *S. suis*

**Fig 1:**
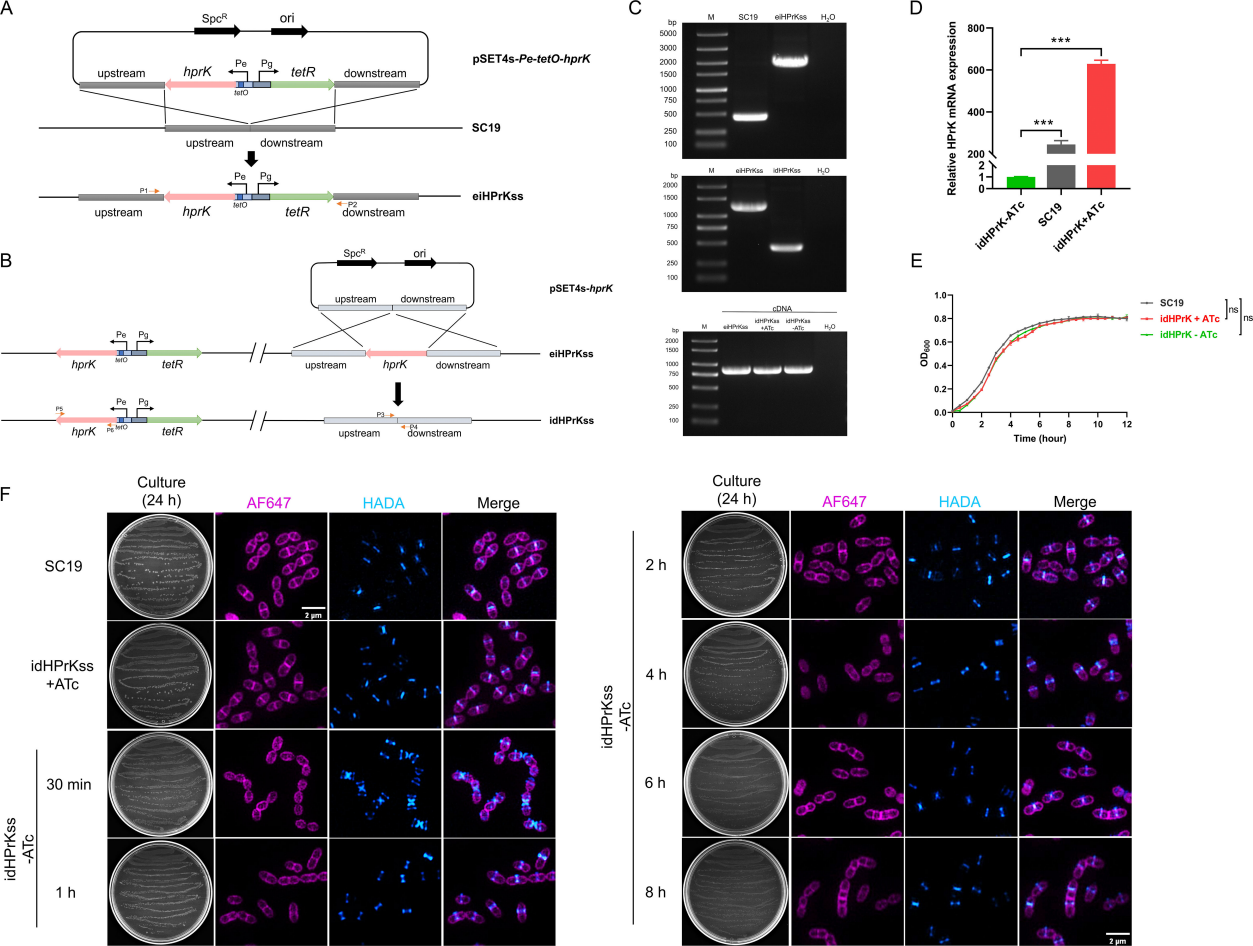
Construction and validation of the ATc-inducible HPrK-depletion strain in *S. suis* (idHPrKss). (**A**) Schematic diagram of the engineered eiHPrKss. The Pg promoter drove the transcription of *tetR*, and the Pe promoter drove the transcription of *hprK*. The operator *tetO* was inserted between Pe and *hprK*. The ATc-inducible *hprK* (Pe-*tetO-hprK* and Pg-*tetR* cassette) was integrated into the genome of *S. suis* SC19 at nucleotide position 1,452,614 through homologous recombination. (**B**) Schematic diagram of the idHPrKss derived from eiHPrKss. The original *hprK* gene was knocked out from eiHPrKss using homologous recombination. (**C**) Identification of the eiHPrKss and idHPrKss using PCR and RT-PCR, respectively. The primers are marked in the above schematic diagrams. To confirm the insertion of the Pe-*tetO-hprK* and Pg-*tetR* cassette, the fragment was detected by PCR using primers P1 and P2. To confirm the knockout of the original *hprK* gene, the fragment was detected by PCR using primers P3 and P4. The transcription of *hprK* was detected by RT-PCR using primers P5 and P6. (**D**) Relative *hprK* transcript levels in SC19 and idHPrK (+/− ATc) strains detected by qRT-PCR. Statistical significance was determined using Dunnett‘s test (***P < 0.001). (**E**) Growth curve (*n* = 3) of WT, idHPrK (+/− ATc); ns: not significant. (**F**) Growth and morphology characterizations of idHPrKss cells were performed under ATc-depleted condition. Peptidoglycan was stained with HADA, and membranes were stained with AF647.

As shown in Fig. 1A through C, an ATc-inducible *hprK* expression cassette was inserted ectopically in an intergenic region, as previously reported (26, 35) (enabling precise, dose- and time-dependent gene regulation without disrupting baseline bacterial growth), and the original *hprK* was knocked out in *S. suis* SC19, a generated idHPrK strain in which the *hprK* was expressed in the presence of ATc, and the cells exhibited normal growth (Fig. 1F). Surprisingly, under ATc depletion, the colony size decreased (Fig. 1F), and *hprK* expression was still detected in the idHPrK strains (Fig. 1C), albeit at a lower level than in the presence of ATc (Fig. 1D). At the same time, bacterial growth does not seem to be affected (Fig. 1E). Morphological analysis revealed transient cell chain elongation and division defects within 30 min of ATc removal; however, these defects subsequently recovered (Fig. 1F).

### ATc depletion led to inactivation of the HPrK inducible system

**Fig 2:**
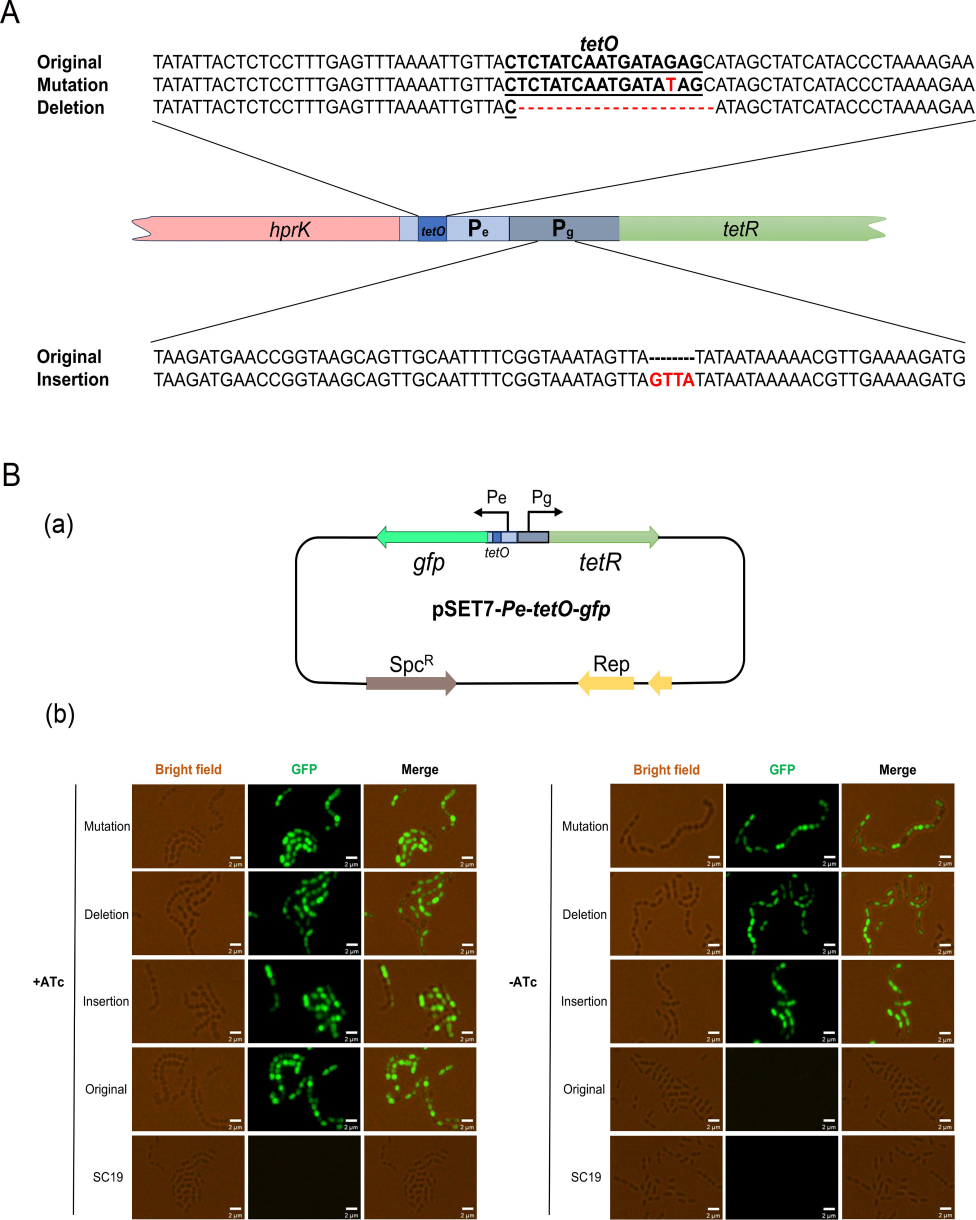
Detection and activity analysis of spontaneous mutations in ATc-inducible expression elements. (**A**) Schematic representation of the wild-type ATc-inducible promoter elements (Original) and three types of spontaneous genetic variations, Mutation, Deletion, and Insertion, in ATc-inducible promoters. (**B**) Activity analysis of the mutants of ATc-inducible promoters. (a) Construction of the GFP reporter systems using ATc-inducible promoter mutants. (b) Observation of GFP expression using a fluorescence microscope. “+ATc” indicates cultures with ATc added, while “−ATc” indicates cultures without ATc.

The colonies of idHPrK strains grown under the ATc depletion condition were selected, and the genomes were sequenced. A total of 3 spontaneous mutation patterns were identified in the AiP elements of these different colonies (Fig. 2A). The dominant mutation was a 17 bp deletion in the *tetO* operator. In addition, a point mutation (G to T) in the *tetO* operator and a 4 bp insertion in the Pg promoter that drives *tetR* were identified. To functionally validate these alterations, the GFP reporter systems driven by these mutated ATc-inducible expression systems were constructed in *S. suis* SC19 (Fig. 2B). Under the absence of the ATc condition, the GFP signal was not detected in the original ATc-inducible system but was detected in the 3 kinds of mutated-inducible systems. This suggested that the alterations in the AiP elements changed the ATc-inducible expression system into a constitutive expression system to ensure *hprK* expression and bacterial survival when ATc was depleted from the medium. This genetic evidence further confirmed that *hprK* is an essential gene for *S. suis*.

### Establishment of an HPrK enzymatic reaction system and screening of inhibitors

**Fig 3:**
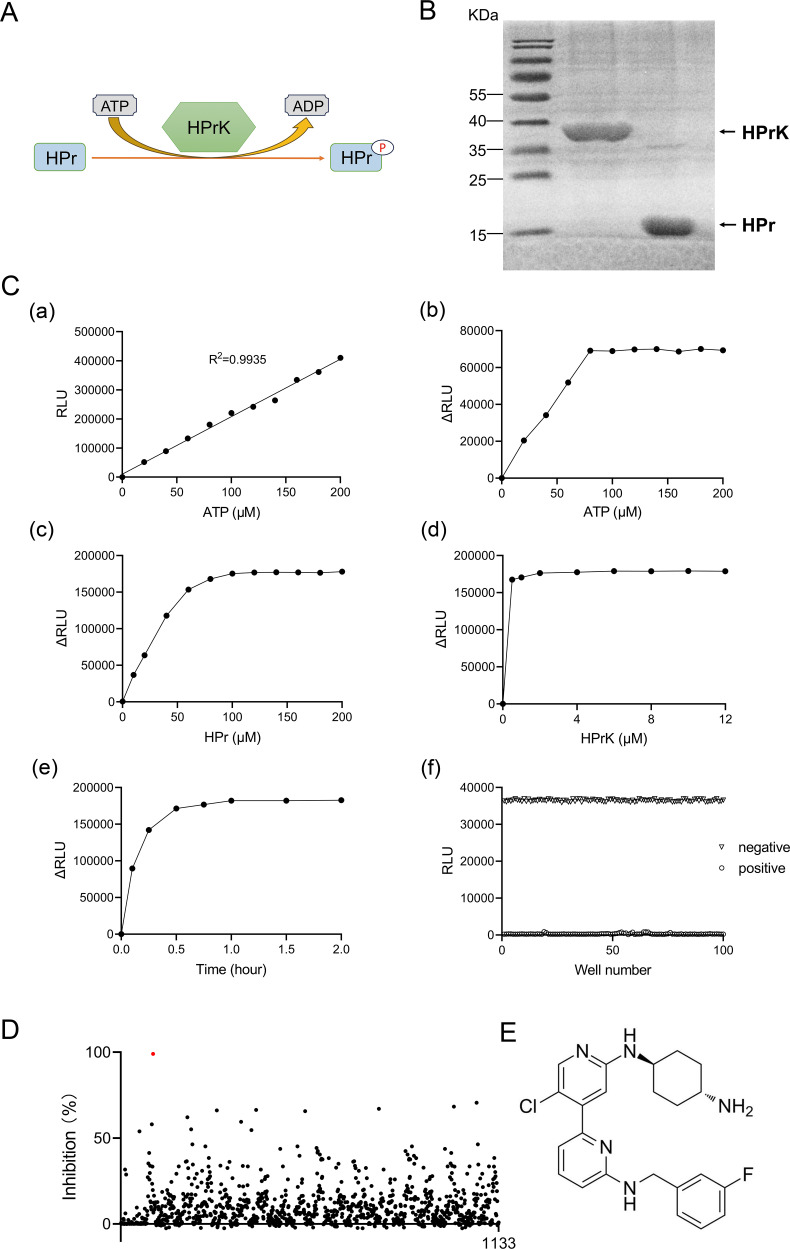
Development of an HPrK enzymatic assay and inhibitor screening. (**A**) Reaction scheme of HPrK-catalyzed phosphorylation. (**B**) SDS-PAGE analysis of purified recombinant HPrK and HPr proteins. (**C**) Optimization and validation of the high-throughput screening assay. (a) Linear correlation between RLU and ATP concentrations. (b–e) Parameter optimization for maximal ΔRLU. ATP, 80 µM; HPr, 100 µM; HPrK, 2 µM; incubation time, 1 h. (f) Quality assessment of testing methods. The assay contained 80 µM ATP, 100 µM HPr, and 2 µM HPrK (positive control) or H_2_O (negative control). The Z’-factor was calculated as 1 − [(3 × SD_N_ + 3 × SD_P_) / (AVG_N_ – AVG_P_)]. SD_P_ and SD_N_ represented the standard deviations among the 100 replicates in the positive and negative control groups, respectively. AVG_P_ and AVG_N_ were the average values of the 100 replicates in the positive and negative control groups, respectively. (**D**) Screening results of the 1,133 compound library. (**E**) Chemical structure of the identified hit compound, CDK9-IN-2 (C₂₃H₂₅ClFN₅).

A HTS assay for HPrK inhibitors was established based on ATP depletion during the reaction (Fig. 3A), and a Kinase-Glo luminescence assay was used to quantify the ATP levels. A good linear correlation between ATP concentration and luminescence intensity was observed over the range of 0–200 μM ATP (Fig. 3C[a]). Optimization was guided by maximizing the difference in relative luminescence units (ΔRLU). The optimized conditions were determined to be 80 μM ATP, 100 μM HPr, and 2 μM HPrK, at 37°C for 1 h (Fig. 3C[b]–[e]). A Z′-factor of 0.82 (Fig. 3C[f]) confirmed the suitability of this assay for HTS.

A total of 1,133 compounds were screened for HPrK inhibitory activity (Fig. 3D). Finally, 13 compounds exhibited an inhibition rate not less than 50%. Among them, the CDK9-IN-2 compound displayed approximately 99% inhibition rate, which was the most potent inhibitor for HPrK kinase activity (Fig. 3D and E).

### Binding mode analysis of CDK9-IN-2 and HPrK

**Fig 4:**
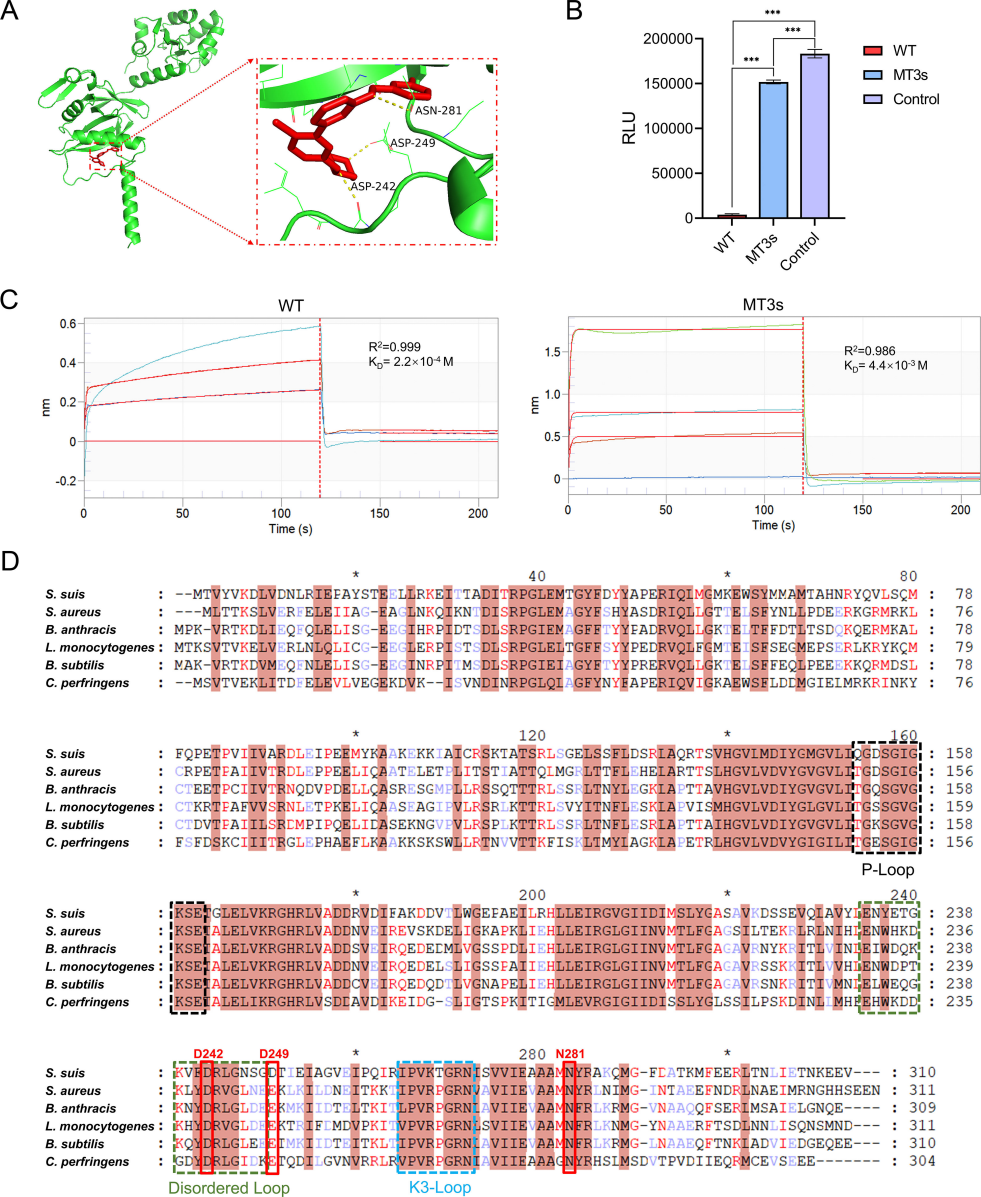
Molecular interaction analysis between CDK9-IN-2 and HPrK. (**A**) Molecular docking results showed that CDK9-IN-2 (red) bound to the residues of the HPrK active site (Asp 242, Asp 249, and Asn 281). (**B**) Enzymatic activity of HPrK mutants assessed by luminescence assay *in vitro*. Statistical significance was determined using Tukey’s test (***P < 0.001). WT, wild-type HPrK; MT3s, the residues of HPrK active site (Asp 242, Asp 249, and Asn 281) were substituted with alanine in HPrK; Control, the reaction mixture lacked HPrK kinase. (**C**) Binding kinetics of CDK9-IN-2 to wild-type HPrK and MT3s measured by biolayer interferometry. The compound concentrations were 0, 32, 64, and 128 μg/mL. (**D**) Multiple sequence alignment of bacterial HPrK orthologs. P-Loop (black box), Disordered-Loop (cyan box), and K3-Loop (blue box). Residues involved in CDK9-IN-2 interaction are marked in a red box.

Three key amino acid residues on HPrK protein, Asp242, Asp249, and Asn281, were recognized as the potential interaction sites of CDK9-IN-2 by molecular docking (Fig. 4A). To further confirm the key binding sites, the three amino acids were substituted with alanine in HPrK protein. BLI assays showed that CDK9-IN-2 binding affinity to the mutated HPrK protein (designated as MT3s) was approximately 20-fold decreased by comparing with wild-type HPrK (designated as WT) (Fig. 4C). In addition, we found that the kinase activity of MT3s decreased by 5-fold (Fig. 4B). These results further confirmed the binding pattern of the inhibitor and enzyme and the inhibition activity of CDK9-IN-2.

It has been reported that three key domains, P-loop, K3-loop, and Disordered-loop, were crucial to the catalytic activity of HPrK (36). Multiple sequence alignment showed that the three key binding sites were located in the Disordered-loop region (Fig. 4D), which played a critical role in dynamic enzyme-substrate interactions. More importantly, these three residues targeted by CDK9-IN-2 were conserved in the HPrK from most gram-positive bacteria (Fig. 4D). Therefore, CDK9-IN-2 might be a broad-spectrum inhibitor for gram-positive bacteria.

### Broad-spectrum antibacterial activity of CDK9-IN-2

The antimicrobial activities of CDK9-IN-2 against gram-negative and gram-positive bacteria, as well as *Mycoplasma* species, were evaluated. Eight clinical multidrug-resistant *S. suis* strains were also used. The MICs of CDK9-IN-2 to tested gram-positives ranged from 16 to 32 μg/mL, and the MBCs ranged from 32 to 64 μg/mL. Consistent inhibitory activity was also observed in the clinical multidrug-resistant *S. suis* strains. In contrast, its MICs and MBCs to tested gram-negatives were equal to or greater than 128 μg/mL (Table 1). This indicated that CDK9-IN-2 had a better antibacterial activity to the gram-positives than the gram-negatives that lack HPrK. In addition, CDK9-IN-2 also showed a similar antibacterial activity to *Mycoplasma* species (Table 1), which also harbor HPrK as gram-positives (37, 38). These results not only supported a target-specific antibacterial mechanism of CDK9-IN-2 but also further confirmed that HPrK is a valuable antimicrobial target.

**TABLE 1:** CDK9-IN-2 antimicrobial activity against different bacteria^*a*^

Bacterial strain	MIC (μg/mL)	MBC (μg/mL)
*S. suis* SC19	16	32
*S. suis* LXJ	16	32
*S. suis* 240121-V-GJ1	16	32
*S. suis* 2403011-T11-GJ2	16	32
*S. suis* 202324-M-GJ2	16	32
*S. suis* L240729-7	16	32
*S. suis* Y240622-3	16	32
*S. suis* Y240622-7	16	32
*S. suis* B240730-1	16	32
*S. aureus* ATCC29213	16	32
*S. aureus* 1213M4A	16	32
*S. pneumoniae* D39	16	32
*S. haemolyticus* Z240617	32	64
*E. faecalis* ATCC29212	32	64
*E. faecium* Y240622	16	32
*E. rhusiopathiae* 1219	32	64
*L. monocytogenes* ATCC19115	32	64
*B. subtilis* WB800N	16	32
*M. gallisepticum* MC3	32	NT
*M. synoviae* 3P4	32	NT
*M. hyorhinis* CVCC361	16	NT
*M. hyopneumoniae* RD2408	16	NT
*E. coli* ATCC25922	128	>128
*S. Typhimurium* ATCC14028	128	>128

^*a*^ MIC, minimum inhibitory concentration; MBC, minimum bactericidal concentration; NT, not tested.

### Antimicrobial efficacy in the *Galleria mellonella* larval infection model

**Fig 5:**
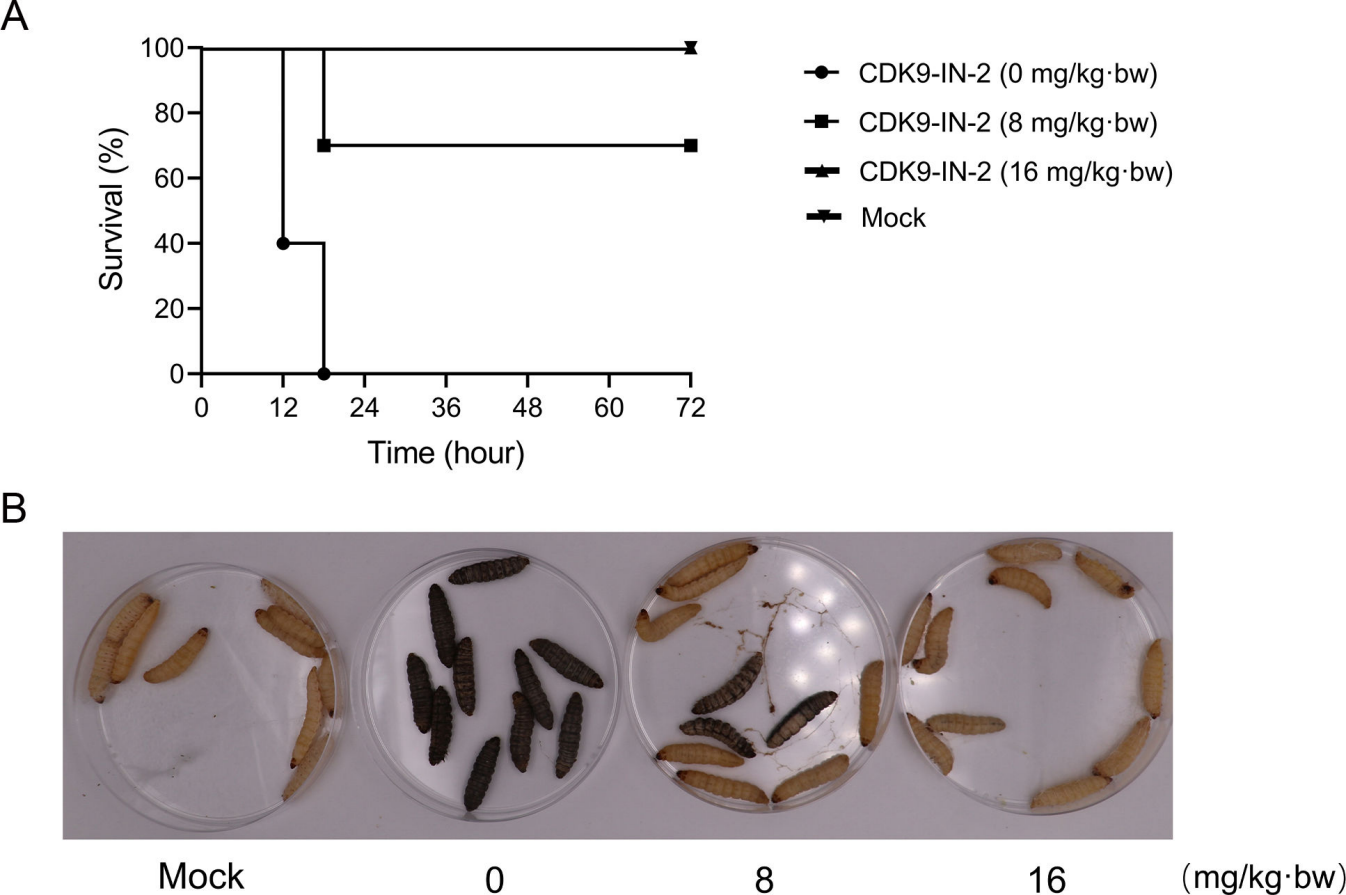
Evaluation of CDK9-IN-2 antimicrobial activity against *S. suis* infection in a *G. mellonella* larval model. (**A**) Survival curve of *G. mellonella* larvae infected with *S. suis* treated with CDK9-IN-2. (**B**) Representative images of *G. mellonella* larvae at 24 h post-infection.

The therapeutic efficacy of CDK9-IN-2 to *S. suis* infection was further assessed using a *G. mellonella* larval infection model. As shown in Fig. 5, all the larvae were dead at 18 h post-infection (hpi), whereas the survival rates of larvae in 2 CDK9-IN-2 treated groups (16 mg/kg and 8 mg/kg) were 100% and 70%, respectively. This result indicated that CDK9-IN-2 may be a potential antibacterial drug for *S. suis* infection.

## DISCUSSION

As stated in the Introduction section, carbon metabolism can be potential target pathways for the development of novel antibacterial agents. Usually, most bacteria can utilize various carbohydrates as carbon sources, but the utilization efficiency is different, so precise regulation of carbon uptake and utilization is particularly important. CCR is one of the most important regulatory mechanisms of carbon metabolism in bacteria. In gram-positive bacteria, HPrK, HPr, and CcpA compose the key regulatory system of CCR, and the phosphorylation/dephosphorylation activity of HPrK acts as the regulatory switch (14, 17, 18). This regulation ensures the utilization of preferred carbon sources for optimized metabolism and the expression of virulence genes (39–42). Thus, HPrK should be a key player in the regulation of carbon metabolism and virulence in gram-positive bacteria. Previous studies have demonstrated that *hprK* deletion causes severe growth defects in some bacteria such as *S. xylosus* and *B. subtilis* (23, 24), while it seems unable to be deleted in *S. pneumoniae* (25) and *S. suis* (Fig. S1). In addition, many *mycoplasma* species also harbor an *hprK* gene (37, 38), but research on its function is relatively scarce. This study first demonstrated that HPrK is essential in *S. suis*, suggesting its potential as an antibacterial drug target. It also identified a broad-spectrum HPrK inhibitor effective against most gram-positives and mycoplasmas *via* high-throughput screening.

In *S. suis*, HPrK has been found to play a crucial role in growth and virulence regulation (22). Under glucose-rich conditions, HPrK promotes the formation of P-HPr-CcpA complex, thus suppressing alternative carbon source metabolism (e.g., galactose and maltose) to optimize energy utilization and promote bacterial growth in both glucose and mixed carbon environments. Furthermore, HPrK modulates pathogenicity by regulating capsular polysaccharide (CPS) biosynthesis and key virulence factors (including *sly*, *eno*, and *cps2a*) (41, 42). In this study, the spontaneous inactivation of the HPrK-inducible system under ATc depletion condition was observed. qRT-PCR confirmed that HPrK expression remained low (Fig. 1D), yet *S. suis* growth was maintained (Fig. 1E). This finding is consistent with previous reports on *B. subtilis* (43) and provides strong experimental evidence for the essential role of HPrK in *S. suis* survival.

Structural analyses reveal remarkable conservation of HPrK across *S. suis* and other gram-positive bacteria, as well as mycoplasmas (Fig. 4D). The protein forms a stable, D_3_-symmetry hexamer through C-terminal helix (α3)-mediated interactions, enhancing enzymatic stability and HPr phosphorylation efficiency. Key functional domains, including the conserved P-loop [(G/A)xxxxGK(S/T)], K3-loop, and Disordered-loop, ensure precise phosphate transfer and dual-activity regulation by modulating ATP binding and conformational switching (36, 37, 44, 45). Functionally, HPrK regulates carbon metabolism and virulence in *S. suis* and other gram-positive bacteria through the P-HPr-CcpA complex. This complex governs critical virulence factors, such as neuraminidases (*nanA*/*nanB*) and β-galactosidase (*bgaA*) in *S. pneumoniae* (40), hemolysin S (*sagA*) in *S. pyogenes* (39), and hemolysins/proteases in *S. aureus* (46). In CcpA-deficient mycoplasmas, HPrK directly regulates metabolism and virulence via HPr-Ser-P and further influences processes including adhesion protein-mediated host cell attachment (44) and biofilm formation (47). This structural and functional conservation underscores HPrK’s potential as a broad-spectrum antimicrobial target.

As previously discussed, HPrK regulates metabolism and virulence factor expression through phosphorylation of the HPr protein. Therefore, kinase activity is essential for its biological function (48, 49). In this study, we developed a luciferase-based ATP consumption assay to screen for HPrK kinase inhibitors and successfully identified CDK9-IN-2 as a potent inhibitor from a library of multitarget kinase inhibitors. Interestingly, although CDK9-IN-2 was originally designed as a eukaryotic CDK9 inhibitor (50), it exhibited remarkable broad-spectrum antibacterial activity against gram-positive bacteria and mycoplasmas. CDK9-IN-2 demonstrated potent efficacy against both standard and clinically resistant strains of *S. suis*, with an MIC of 16 μg/mL. It represented a 4- to 25-fold improvement over traditional plant-derived antimicrobials, such as forsythiaside (64 μg/mL) and paeoniflorin (400 μg/mL) (51, 52). The therapeutic potential of CDK9-IN-2 was further validated *in vivo*, where it significantly improved survival outcomes in a *G. mellonella* infection model. Although CDK9-IN-2 showed weak antibacterial activity against *E. coli* and *Salmonella* Typhimurium (MIC = 128 µg/mL), suggesting potential additional targets in these gram-negative bacteria, its activity is substantially lower than that against HPrK-possessing bacteria (MIC = 16 µg/mL). This underscores HPrK as the primary target of CDK9-IN-2. Notably, CDK9-IN-2 was initially screened as a cancer cell inhibitor, which has a significant inhibitory effect on cancer cells and may exhibit toxicity to mammalian cells (53). Therefore, our next steps will involve a comprehensive safety evaluation (e.g., acute, subacute, and chronic toxicity in mammals) and potentially its structural optimization to fully assess its pharmacological potential.

In this study, molecular docking and BLI analysis demonstrated that the small-molecule inhibitor CDK9-IN-2 primarily targets the Disordered-loop region of *S. suis* HPrK. Key binding residues include Asp242, Asp249, and a previously unreported conserved site, Asn281. Previous research has predominantly focused on the P-loop and K3-loop, but the conformation of the Disordered-loop region is highly dynamic, and its mechanistic details remain incompletely understood (36, 45). Our findings reveal that direct binding of CDK9-IN-2 to this region significantly suppresses kinase activity and bacterial growth, underscoring the functional importance of its conformational dynamics in substrate binding and enzymatic function. We speculate that CDK9-IN-2-mediated inhibition of HPrK leads to HPr in a dephosphorylated state, thereby disrupting CCR and impairing bacterial energy metabolism. Notably, while residue 249 of HPrK varies between bacterial species (Asp or Glu), these residues are structurally and functionally conserved and can be interchanged without loss of activity (54). Asp242 and Asn281 also exhibit high conservation across diverse bacterial species. Furthermore, we employed a strategy combining standard strains (e.g., *S. aureus* ATCC 29213 and *S. pneumoniae* D39) with clinical isolates (e.g., *S. haemolyticus* Z240617) (Table S1) to systematically evaluate the antibacterial activity of CDK9-IN-2. This approach ensured both experimental reproducibility and clinical relevance. The results demonstrated that CDK9-IN-2 exhibited significant inhibitory activity against various pathogens (Table 1), suggesting broad-spectrum antibacterial potential. To further define its antibacterial spectrum, subsequent studies will include a broader range of clinical strains.

In conclusion, our study established that the serine kinase HPrK is an essential kinase in *S. suis* and functions as a promising broad-spectrum antibacterial target. Based on this target, an effective compound inhibitor has been screened out with broad-spectrum antibacterial activity against multidrug-resistant *S. suis* isolates as well as diverse gram-positives and mycoplasmas. The binding sites between the inhibitor and target are conserved among the pathogenic bacteria. These findings provide novel approaches for developing HPrK-targeted broad-spectrum antimicrobial agents.

## Data Availability

The data sets generated and/or analyzed during the current study are available from the corresponding author on reasonable request.

## SUPPLEMENTAL MATERIAL

Supplemental material spectrum.03977-25-s0001.pdf Fig. S1 and S2; Tables S1 to S3.

ASM does not own the copyrights to Supplemental Material that may be linked to, or accessed through, an article. The authors have granted ASM a non-exclusive, world-wide license to publish the Supplemental Material files. Please contact the corresponding author directly for reuse.
