# Supplementary material for: Evaluation of serine protein kinase HPrK as an antibacterial target in gram-positive bacteria and mycoplasmas
Source: Microbiol Spectr. 2026 Mar 17;14(4):e03977-25. doi: 10.1128/spectrum.03977-25 (PMC13055401; doi:10.1128/spectrum.03977-25)
Supplement: Supplemental material — Fig. S1 and S2; Tables S1 to S3. [file spectrum.03977-25-s0001.pdf]

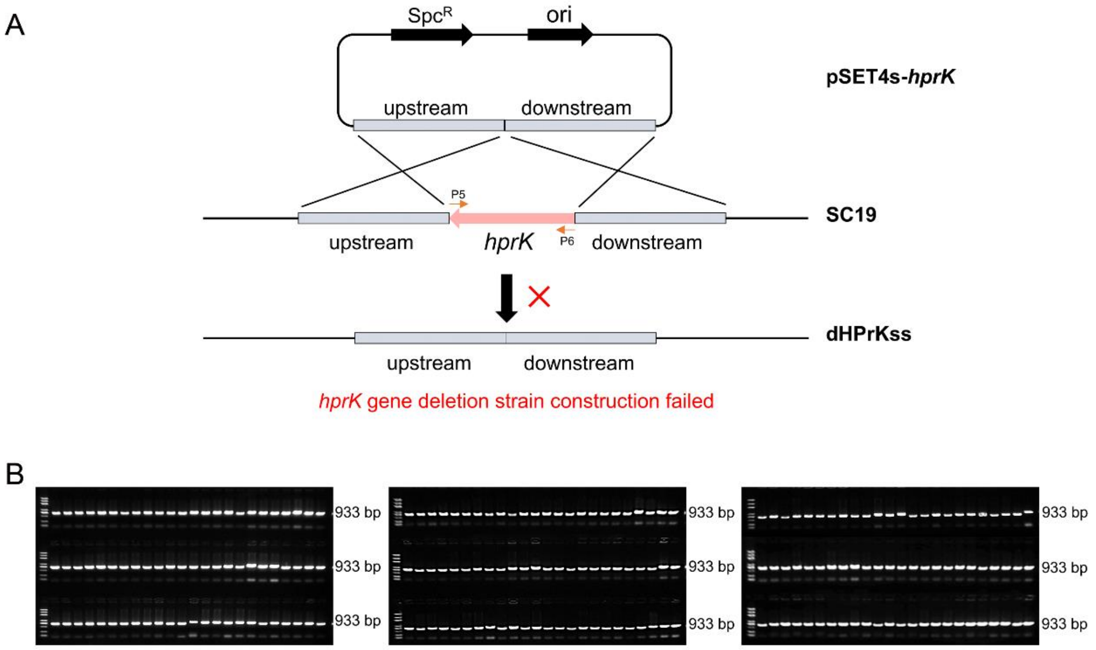

Figure S1. Generation of *hprK* deletion strain of *S. suis via* traditional homologous recombination. (A) Schematic representation of the strategy to delete *hprK* gene using the temperature-sensitive plasmid pSET4s-*hprK*. **(B)** Identification of *hprK* deletion by PCR with primers P5 and P6. The 933 bp fragment of the *hprK* gene remained detectable in all selected colonies, indicating that the *hprK* deletion strain was not obtained.

(A)

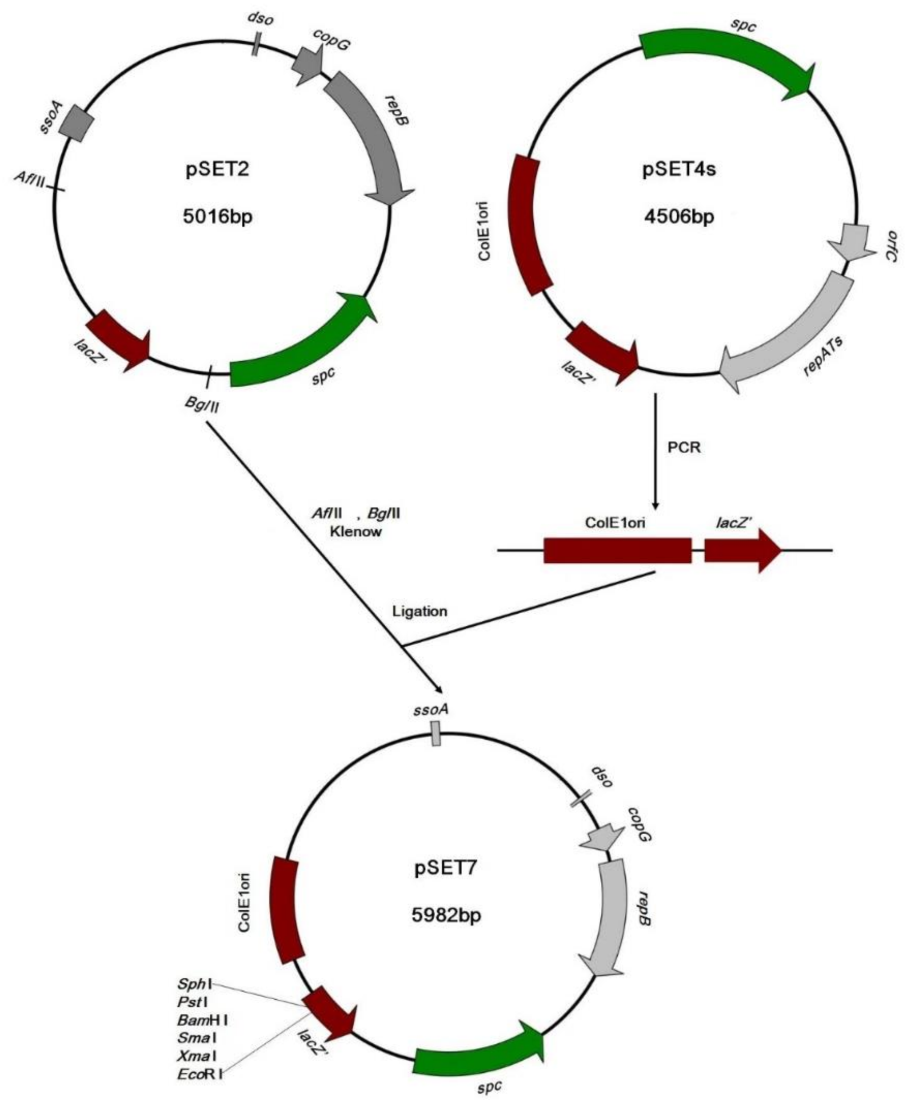

(B) Sequence of pSET7

1 ATTAATTTTG AAATATTAAA TATTTTTGGA TATATAGGTG TTAAGTTTGA AATTATTACC
61 GCTTATGAAA ATGGGAAATT AGTTGAAACG CAAATTATTG TGGATGACAT AGAGTTGATA
121 AAAGTTGAAA AATTTTTTGA TTAAAGAGAA GAAGAGGGGG CGTACTACGA CCCCCCTTTT
181 AAGTGCCGAG TGCCAAATTG CTAAAAAAAG TGCCGTAAAG CTAGATACAT CAAGGGTTTA
241 GCTCATGCTA CACTTCGGCG ACATTTTGGC GACATTTTCG GCGACAAAAA GAAAAAAATA
301 CGATAAATGT TGCAATGTAT GCACAGTTAT GCTACTATGG TTTTATAAAT TTTTGAGAGG
361 TGGAACATGA AAAAAAGATT GACGATAACA CTCAGTGAAT CGGTGCTTGA AAATCTTGAA
421 AAAATGGCAA AAGAGATGGG GTTGTCAAAA TCTGCACTGA TTTCAGTTGC CTTGGAAAAC
481 TACAAGAAAG GTCAAGTAAA ATAAAAAAAA GCCGAGCTGG CAGGCGGGCT TTTAAAAGTA
541 TTATAACTTG GGTTAATTAT AACTTATGGC TAAAGAAAAA GCAAGATACT TCACTTTTTT
601 ACTCTATCCT GAGTCAATCC CTAGTGATTG GGAATTGAAA TTAGAATTGC TGGGCGTACC
661 TATTGCAGTC AGCCCTTTGC ATGATAGAGA CAAGAGTGAT GTTGAGGGAC AACAATATAA
721 AAAACCGCAC TATCATGTGA TTTATGTTTC AAAAAATCCA GTCACGGCAG ATAGTGTGAG
781 AATGAAAATT AAGAGGTCAC TTGGAGATAA TAGCGTTGCT CTAGTTCAGA TAATTAGAAC
841 TAGCATTGAG AACACGTATT TATATCTTAC GCATGAATCA AAGGACGCTA TCGAAAAGAA
901 AAAGCACGTC TATGATAAGG CTGACATAAC TTTGCTTAGT AACTTTGATA TAGACCGTTA
961 TATAACGCTT GACGTCGAAG AGAAAGACGA TATGCTCAAT GAGGTTTGTG ACCTTATTGA
1021 TGAGTATGAT ATTGCAAATA TGCGTGAATT GAGACGTTTT ATTAAACTTC ATGGGGCTGA
1081 ACATGGTTTA CCTAGTATCA AAATTATAAA TTCAGTTTTA CGTTCGCATA CTGGGTTAAT
1141 ACGTCTGTAT TTTGATGCTG TCTATCAAGA ACGTAAGTAT GGCAGTAATA TCCCTAATGT
1201 TAATCAAGAG ACAGGCGAGA TTTTAAGTGA GGATAGCGAT GACTGATAGA AATGAGCTGA
1261 TTAACGATAT TGCAGAGTTG AAAGCAAAAA GAGACAGACT ACTGGCTCAA ATGAAAGAGG
1321 CGGAACAATG GGAAAGCGTA GCGTGGGATA GTTACTATGC AGTCGCAGAC CATGTGAAAG
1381 CTCTTGAGAA AAAGCAGGAG ATAGGTCGAA ACTATTGGGA AAGTTCTCAA AGAGCTATTA
1441 GTCATCAATT TGACTTTGTG GCTGACCAAG CTAACAAAGT CAAAAAGGTG TTGGCTAAAA

1501 AGAGATATGA TTTGCTAGAT GAGGAAATCG ACAAGCTGAT GAACGAAGTT CGAGAGCTGG
1561 CAGATGTCCT TGGGATTGAG ATTGACGAGC TCGGTTTATT GTTTTCTAAA ATCTGATTAC
1621 CAATTAGAAT GAATATTTCC CAAATATTAA ATAATAAAAC AAAAAAATTG AAAAAAGTGT
1681 TTCCACCATT TTTTCAATTT TTTTATAATT TTTTTAATCT GTTATTTAAA TAGTTTATAG
1741 TTAAATTTAC ATTTTCATTA GTCCATTCAA TATTCTCTCC AAGATAACTA CGAACTGCTA
1801 ACAAAATTCT CTCCCTATGT TCTAATGGAG AAGATTCAGC CACTGCATTT CCCGCAATAT
1861 CTTTTGGTAT GATTTTACCC GTGTCCATAG TTAAAATCAT ACGGCATAAA GTTAATATAG
1921 AGTTGGTTTC ATCATCCTGA TAATTATCTA TTAATTCCTC TGACGAATCC ATAATGGCTC
1981 TTCTCACATC AGAAAATGGA ATATCAGGTA GTAATTCCTC TAAGTCATAA TTTCCGTATA
2041 TTCTTTTATT TTTTCGTTTT GCTTGGTAAA GCATTATGGT TAAATCTGAA TTTAATTCCT
2101 TCTGAGGAAT GTATCCTTGT TCATAAAGCT CTTGTAACCA TTCTCCATAA ATAAATTCTT
2161 GTTTGGGAGG ATGATTCCAC GGTACCATTT CTTGCTGAAT AATAATTGTT AATTCAATAT
2221 ATCGTAAGTT GCTTTTATCT CCTATTTTTT TTGAAATAGG TCTAATTTTT TGTATAAGTA
2281 TTTCTTTACT TTGATCTGTC AATGGTTCAG ATACGACGAC TAAAAAGTCA AGATCACTAT
2341 TTGGTTTTAG TCCACTCTCA ACTCCTGATC CAAACATGTA AGTACCAATA AGGTTATTTT
2401 TTAAATGTTT CCGAAGTATT TTTTTCACTT TATTAATTTG TTCGTATGTA TTCAAATATA
2461 TCCTCCTCAC TATTTTGATT AGTACCTATT TTATATCCAT AGTTGTTAAT TAAATAAACT
2521 TAATTTAGTT TATTTATAGA TTTCATTGGC TTCTAAATTT TTTATCTAGA TAATAATTAT
2581 TTTAGTTAAT TTTATTCTAG ATTATATATG ATATGATCTT TCATTTCCAT AAAACTAAAG
2641 TAAGTGTAAA CCTATTCATT GTTTTAAAAA TATCTCTTGC CAGTCACGTT ACGTTATTAG
2701 TTATAGTTAT TATAACATGT ATTCACGAAC AGATCTCGGT GATGACGGTG AAAACCTCTG
2761 ACACATGCAG CTCCCGGAGA CGGTCACAGC TTGTCTGTAA GCGGATGCCG GGAGCAGACA
2821 AGCCCGTCAG GGCGCGTCAG CGGGTGTTGG CGGGTGTCGG GGCTGGCTTA ACTATGCGGC
2881 ATCAGAGCAG ATTGTACTGA GAGTGCACCA TATGCGGTGT GAAATACCGC ACAGATGCGT
2941 AAGGAGAAAA TACCGCATCA GGCGCCATTC GCCATTCAGG CTGCGCAACT GTTGGGAAGG
3001 GCGATCGGTG CGGGCCTCTT CGCTATTACG CCAGCTGGCG AAAGGGGGAT GTGCTGCAAG
3061 GCGATTAAGT TGGGTAACGC CAGGGTTTTC CCAGTCACGA CGTTGTAAAA CGACGGCCAG
3121 TGAATTCGAG CTCGGTACCC GGGGATCCTC TAGAGTCGAC CTGCAGGCAT GCAAGCTTGG
3181 CGTAATCATG GTCATAGCTG TTTCCTGTGT GAAATTGTTA TCCGCTCACA ATTCCACACA
3241 ACATACGAGC CGGAAGCATA AAGTGTAAAG CCTGGGGTGC CTAATGAGTG AGCTAACTCA
3301 CATTAATTGC GTTGCGCTCA CTGCCCGCTT TCCAGTCGGG AAACCTGTCG TGCCAGCTGC
3361 ATTAATGAAT CGGCCAACGC GCGGGGAGAG GCGGTTTGCG TATTGGGCGC TCTTCCGCTT
3421 CCTCGCTCAC TGACTCGCTG CGCTCGGTCG TTCGGCTGCG GCGAGCGGTA TCAGCTCACT
3481 CAAAGGCGGT AATACGGTTA TCCACAGAAT CAGGGGATAA CGCAGGAAAG AACATGTGAG
3541 CAAAAGGCCA GCAAAAGGCC AGGAACCGTA AAAAGGCCGC GTTGCTGGCG TTTTTCCATA
3601 GGCTCCGCCC CCCTGACGAG CATCACAAAA ATCGACGCTC AAGTCAGAGG TGGCGAAACC
3661 CGACAGGACT ATAAAGATAC CAGGCGTTTC CCCCTGGAAG CTCCCTCGTG CGCTCTCCTG
3721 TTCCGACCCT GCCGCTTACC GGATACCTGT CCGCCTTTCT CCCTTCGGGA AGCGTGGCGC
3781 TTTCTCAATG CTCACGCTGT AGGTATCTCA GTTCGGTGTA GGTCGTTCGC TCCAAGCTGG
3841 GCTGTGTGCA CGAACCCCCC GTTCAGCCCG ACCGCTGCGC CTTATCCGGT AACTATCGTC
3901 TTGAGTCCAA CCCGGTAAGA CACGACTTAT CGCCACTGGC AGCAGCCACT GGTAACAGGA
3961 TTAGCAGAGC GAGGTATGTA GGCGGTGCTA CAGAGTTCTT GAAGTGGTGG CCTAACTACG
4021 GCTACACTAG AAGGACAGTA TTTGGTATCT GCGCTCTGCT GAAGCCAGTT ACCTTCGGAA
4081 AAAGAGTTGG TAGCTCTTGA TCCGGCAAAC AAACCACCGC TGGTAGCGGT GGTTTTTTTG
4141 TTTGCAAGCA GCAGATTACG CGCAGAAAAA AAGGATCTCA AGAAGATCCT TTGATCTTTT
4201 CTACGGGGTC TGACGCTCAG TGGAACGAAA ACTCACGTTA AGGGATTTTG GTCATGAGAT
4261 TATCAAAAAG GATCTTCACC TAGATCCTTT TAAATTAAAA ATGAAGTTTT AAATCAATCT
4321 AAAGTATATA TGAGTAAACT TGGTCTGACA GTTACCAATG CTTAATCAGT GAGGCACCTA
4381 TCTCAGCGAT CTGTCTATTT CGTTCATCCA TAGTTGCCTG ACTCCCCGTC GTGTAGATAC
4441 TTAAGGAAAA ATACGAGGAG TTAGACAAAA TCGAGCTACG AGCCTCTGAG AGCCTCTCAG
4501 AGCTATCTGG TGTCGAAGGG TATATAAATA ACCTAGAAAA ACATTCAAGC GCCTTAGAAA
4561 GCAAAATAGG GGGCTTAGAA AACGAATATT TAAGACTGGC TAAACAAAAT GCCCGATTAA
4621 GCGACTTGAA AATCATGTCT GAAAAAGAAT TGGCTGAAAT TCAGCCGAAA AAAGGCATGT
4681 TTGGAAAAGA ACATGTCGAA TTGACTAAAG AGCAATTTGA GAAATTTAAG GGATTGATAT
4741 ACCGAAGCAA GAATTTAGTT CATGAAAAGG AATTAGAGAA TGACCAACTA AGGCGGCAAA
4801 TGCCTCTGAG AAGCTCAAAA AACGGTTTTG AGGCGAGTTT GCAACGGGCT AAGGATAAAA
4861 CTAAAGGAGA AAGCATAGAC CGCCTTAAAA GCGAAATTAG AGGGCTTAAA AATGAAAACT
4921 CGGTTTTGAG ACAGCAAAAT GACAAAATGC TAGGTAAGTT AAGAGAGTTT ATGCCTGATA
4981 AAGCGTTAAA TAATTTTATT TCTGAACTTA AATCAATAAA ACCTATCGTG AAGATAGTTA
5041 AAAGAGTTAT TGAAAAAGGC TTAGGGCTCT AAGCGATTTA TGCCGAGAAA ACTCTTGCTA
5101 GGAAGCTATG CGAAATAGAC TAGTAGGGGT CGAGTCGACA GGCTGAAAGC TTGCCGACCG
5161 AACACGACAG TCAGATTTCA GCATAGGTTT CTTCCTAGCA AGAGGAAATT GGAATAAAGA
5221 AAGCGTATCA AAGTAAAACT AAAAGGACTA AGTCCTGTGT AATACAGGCC TTAGTCCTTT
5281 ACTTAAAATA GAATGTCCAA CTTTTTGGGG TCAGTATATA TTTTAGTCTT TTTTATTCTA
5341 TAGTTGGGAT AGAGATTTGT ACTCCTCCTG CGCCATATGC AATTCCGCTT ATACCTGCAT
5401 TTCTTGCTTT TTTAGGTAAA CCGAATGAAC CACTAACTGA TTGACCTCCT TTGCCAGATT
5461 TGCTGACAGG ATATATTCTA GTAGTGTTAT CTGAGAAAGT AACTGTTATC AATCCAGAAT
5521 ATGTATAACT TTTTGATGTA TTAGGTTTAA CTGACCACCA AACTTCTCGA CCACCACCAT
5581 ATAATTCGAT TTTTGTATAT CCGCCATTTC CGTAACTTGT TTTGTAAGGA ACTATTTCAC
5641 CGGTATAAGA ATAATCAGTT GTAGTTGAAA TATTTGTATT TGAGGTAGTG ACTGTATTAT
5701 CTGCAAAAAC AGTAGAAATG GATATAAATC CTAACATAGA AAAAAGAGTG ACAAATACAA
5761 TTATTGATTT TTTGAAACTT TTCATGAAAG TTTACCTCCG TTTTTTTGTT ATAATTATAG
5821 CAAAAAAATA ATTAAAAAGA AAGCGTTATC ATAGAAATGA AAAAGAATTT TATTGCAATT
5881 ATAAAAAACA TATTAAAAAA CAAAAATATC CCTAAATATT GTTTTGAAAT TATTGAAAAA
5941 GATGGAAGTA CTTGTTTTCT TTCGACAAAT TACTTTGATG TA
//

Figure S2. Construction of plasmid pSET7. (A) Schematic diagram of the strategy to construct pSET7 from pSET2. The *lacZ'* fragment in pSET2 is replaced by the fragment *ColE1 ori-lacZ'* from pSET4s. **(B)** The DNA sequence of pSET7.

Table S1. Primers used in this study.

Primers name	Primers sequence (5'→3')
4s-*hprK*-F	CCACCTATTCCAAGGAATTATTAAGCCTCTTCTCTATTGTGTTCAAT
4s-*hprK*-R	ACTCAAAGGAGAGTAATATAATGACCGTTTATGTGAAAGATTTGG
4s-UP-F	AAACGACGGCCAGTGAATTCTCATAAATTCGGATGGTTGAAAAA
4s-UP-R	ACAATAGAGAAGAGGCTTAATAATTCCTTGGAATAGGTGGGG
4s-DOWN-F	AATGTGAAAGTGGGTCTTAATAAAGATTAAGTTATTTTATACAC
4s-DOWN-R	GCAGGTCGACTCTAGAGGATCCATACCATGCGGAAAACATTTG
4s-*tetR*-F	TCTTTCACATAAACGGTCATTATATTACTCTCCTTTGAGTTTAAAATTGTTAC
4s-*tetR*-R	ATAAAATAACTTAATCTTTATTAAGACCCACTTTCACATTT
4s-UP_{hprK}-F	AAACGACGGCCAGTGAATTCAAAGAGGATCAAGGGTCGAA
4s-UP_{hprK}-R	CTGAAAGTGAGGGAAGAAAACAGATGGGGTTTGATGC
4s-$DOWN_{hprK}$-F	CATCAAACCCCATCTGTTTTCTTCCCTCACTTTCAGTT
4s-$DOWN_{hprK}$-R	GCAGGTCGACTCTAGAGGATCCACACTGACAAGCAGGTAAG
pET28a-HPr-F	CAAATGGGTCGCGGATCCATGGCTTCAAAAGACTTCC
pET28a-HPr-R	GCCGCAAGCTTGTCGACTTATGCCAATCCTTCTTTTT
pET28a-HPrK-F	AAATGGGTCGCGGATCCATGACCGTTTATGTGAAAG
pET28a-HPrK-R	CGCAAGCTTGTCGACTTAAGCCTCTTCTCTATT
pSET7-GFP-F	ACGACGGCCAGTGAATTCTTATTTGTATAGTTCATCCATGCC
pSET7-GFP-R	CAAAGGAGAGTAATATAATGAGTAAAGGAGAAGAACTT
pSET7-*tetR*-F	TTCTTCTCCTTTACTCATTATATTACTCTCCTTTGAGTTT
pSET7-*tetR*-R	GGTCGACTCTAGAGGATCCTTAAGACCCACTTTCACATTT
P1	TGGTCCACTGGACCATAACT
P2	ATCCCCCCTGGTTTAGAATG
P3	TAGGATAGAAAGCGGAAATG
P4	TTCGTGCATAACAGGTGTG
P5	TTAAGCCTCTTCTCTATTGTG
P6	ATGACCGTTTATGTGAAAGAT
pET28a-$HPrK_{D242A- D249A}$-F	GTAAGGTATTTGCCCGTTTGGGAAACTCTGGCGCTACTATCGAAAT
pET28a-$HPrK_{D242A- D249A}$-R	ATTTCGATAGTAGCGCCAGAGTTTCCCAAACGGGCAAATACCTTAC
pET28a-$HPrK_{N281A}$-F	AGCAGCCATGGCCTACCGTGCCA
pET28a-$HPrK_{N281A}$-R	TGGCACGGTAGGCCATGGCTGCT

Table S2. Plasmids used in this study.

Plasmids name	Description	Reference or source
pSET4s	*S. suis* temperature sensitive suicide vector, resistant to SPC	[1]
pSET2	*S. suis*-*E. coli* shuttle cloning vector, resistant to SPC	[2]
pSET7	*S. suis* expression vector derived from pSET2, resistant to SPC	This study
pET28a	*E. coli* expression vector, resistant to KAN	Novagen
pSET4s-*Pe-tetO-hprK*	Recombinant plasmid for the insertion of *Pe-tetO-hprK* fragment into the ectopic genomic location of *S. suis*, resistant to SPC	This study
pSET4s-*hprK*	Recombinant plasmid for knockout of *hprK* in *S. suis*, resistant to SPC	This study
pSET7-*Pe-tetO-gfp*	GFP reporter plasmid in *S. suis*, resistant to SPC	This study
pET28a-His-HPr	HPr expressing plasmid in *E. coli*, resistant to KAN	This study
pET28a-His-HPrK	HPrK expressing plasmid in *E. coli*, resistant to KAN	This study
pET28a-$HPrK_{D242A\text{-}D249A\text{-}N281A}$	$HPrK_{D242A\text{-}D249A\text{-}N281A}$ expressing plasmid in *E. coli*, resistant to KAN	This study

Table S3. Bacterial strains used in this study.

Bacterial strains	Description	Reference or source
**Engineered strain**		
S. suis SC19	*S. suis* serotype 2, an isolate from a diseased pig	[3]
eiHPrKss	Ectopic ATc-induced HPrK-expressing *S. suis* strain	This study
idHPrKss	ATc-inducible *hprK*-deletion strain of *S. suis*	This study
E. coli DH5α	Host for cloning vector	Kangwei
E. coli BL21(DE3)	Host for expression vector	Kangwei
**Clinical resistant strains**		
S. suis LXJ	Isolation from a diseased pig (Hubei, China), confirmed by PCR [4]. Resistant to TET, TIL	Laboratory stock
S. suis 240121-V-GJ1	Isolation from a diseased pig (Gansu, China), confirmed by PCR [4]. Resistant to TET, TIL, STR	Laboratory stock
S. suis 2403011-T11-GJ2	Isolation from a diseased pig (Yunnan, China), confirmed by PCR [4]. Resistant to TET, TIL, KAN	Laboratory stock
S. suis 202324-M-GJ2	Isolation from a diseased pig (Hunan, China), confirmed by PCR [4]. Resistant to TET, TIL, STR, LIN	Laboratory stock
S. suis L240729-7	Isolation from a diseased pig (Hunan, China), confirmed by PCR [4]. Resistant to TET, TIL, STR, KAN, AMK, LIN, CHL, GEN, FFC	Laboratory stock
S. suis Y240622-3	Isolation from a diseased pig (Hubei, China), confirmed by PCR [4]. Resistant to TET, TIL, STR, KAN, AMK	Laboratory stock
S. suis Y240622-7	Isolation from a diseased pig (Hubei, China), confirmed by PCR [4]. Resistant to TET, TIL	Laboratory stock
S. suis B240730-1	Isolation from a diseased pig (Hunan, China), confirmed by PCR [4]. Resistant to TIL, AMK, LIN, FFC	Laboratory stock
**Spectrum of activity**		
S. aureus ATCC29213	*S. aureus* standard strain	Laboratory stock
S. aureus 1213M4A	MRSA	Laboratory stock
S. pneumoniae D39	*S. pneumoniae* serotype 2, classic virulent strain	Laboratory stock
S. haemolyticus Z240617	An isolate from a diseased pig	Laboratory stock
E. faecalis ATCC29212	*E. faecalis* standard strain	Laboratory stock
E. faecium Y240622	An isolate from a diseased pig	Laboratory stock
E. rhusiopathiae 1219	*E. rhusiopathiae* standard strain	Laboratory stock
L. monocytogenes ATCC19115	*L. monocytogenes* standard strain	Laboratory stock
B. subtilis WB800N	Derivative of *B. subtilis* 168	[5]
M. gallisepticum MC3	An isolate from a diseased pig	Laboratory stock
M. synoviae 3P4	An isolate from a diseased pig	Laboratory stock
M. hyorhinis CVCC361	*M. hyorhinis* standard strain	CVCC
M. hyopneumoniae RD2408	*M. hyorhinis* standard strain	CVCC
E. coli ATCC25922	*E. coli* standard strain	Laboratory stock
S. Typhimurium ATCC14028	*S.* Typhimurium standard strain	Laboratory stock

SPC, spectinomycin; KAN, kanamycin; TET, tetracycline; TIL, tilmicosin; AMK, amikacin; STR, streptomycin; LIN, lincomycin; CHL, chloramphenicol; GEN, gentamicin; FFC, florfenicol; CVCC, National Center for Veterinary Culture Collection;

[1] Takamatsu D, Osaki M, Sekizaki T. 2001. Thermosensitive suicide vectors for gene replacement in Streptococcus suis. Plasmid 46:140-148. doi:10.1006/plas.2001.1532.

[2] Takamatsu D, Osaki M, Sekizaki T. 2001. Construction and characterization of Streptococcus suis-Escherichia coli shuttle cloning vectors. Plasmid 45:101-113. doi:10.1006/plas.2000.1510.

[3] Li W, Liu L, Chen H, Zhou R. 2009. Identification of Streptococcus suis genes preferentially

expressed under iron starvation by selective capture of transcribed sequences. FEMS Microbiol Lett 292:123-133. doi:10.1111/j.1574-6968.2008.01476.x.

[4] Wang L, Qiu J, He B, Wu X, Chen Q, Wang Q, Wu R, Zheng B, Zhou L, Huang X. 2025. Isolation, Identification, and Molecular Genetic Characteristics of a Pathogenic Strain of Streptococcus suis Serotype 3. Pathogens 14:doi:10.3390/pathogens14020192.

[5] Jeong H, Jeong DE, Park SH, Kim SJ, Choi SK. 2018. Complete Genome Sequence of Bacillus subtilis Strain WB800N, an Extracellular Protease-Deficient Derivative of Strain 168. Microbiol Resour Announc 7:doi:10.1128/MRA.01380-18.
